# Scraping Therapy Improved Muscle Regeneration through Regulating GLUT4/Glycolytic and AMPK/mTOR/4EBP1 Pathways in Rats with Lumbar Multifidus Injury

**DOI:** 10.1155/2023/8870256

**Published:** 2023-06-22

**Authors:** Bin Zou, Juan Du, Qiwen Xuan, Yajing Wang, Zixiao Wang, Wen Zhang, Lianghua Wang, Wei Gu

**Affiliations:** ^1^Department of Traditional Chinese Medicine, Naval Medical University, Shanghai 200433, China; ^2^Department of Biochemistry and Molecular Biology, College of Basic Medical, Naval Medical University, Shanghai 200433, China; ^3^Dujiangyan Air Force Special Service Sanatorium, Chengdu 611838, China

## Abstract

**Background:**

High morbidity of nonspecific low back pain (NLBP) and large consumption of medical resources caused by it have become a heavy social burden. There are many factors inducing NLBP, among which the damage and atrophy of multifidus (MF) are most closely related to NLBP. Scraping therapy can have significant treatment effects on NLBP with fewer adverse reactions and less medical fund input than other modalities or medications. However, the mechanism of scraping therapy treating NLBP remains unclarified. Here, we wanted to investigate the effects of scraping therapy on promoting MF regeneration and the underlying mechanisms.

**Methods:**

A total of 54 male rats (SD, 6-7 weeks old) were randomly divided into nine groups, namely, K, M6h, M1d, M2d, M3d, G6h, G1d, G2d, and G3d, with six rats in each group. They were injected with bupivacaine (BPVC) to intentionally induce MF injury. We then performed scraping therapy on the rats that had been randomly chosen and compared treatment effects at different time points. *In vitro* data including skin temperature and tactile allodynia threshold were collected and histological sections were analyzed. mRNA sequencing was applied to distinguish the genes or signaling pathways that had been altered due to scraping therapy, and the results were further verified through reverse transcription polymerase chain reaction and Western blot analysis.

**Results:**

Transitory petechiae and ecchymosis both on and beneath the rats' skin raised by scraping therapy gradually faded in about 3 d. Cross-sectional area (CSA) of MF was significantly smaller 30 h, 2 d, and 4 d after modeling (*P*=0.007, *P*=0.001, and *P*=0.015, respectively, vs. the blank group) and was significantly larger in the scraping group 1 d after treatment (*P*=0.002 vs. the model 1d group). Skin temperature significantly increased immediately after scraping (*P* < 0.001) and hindlimb pain threshold increased on the 2nd day after scraping (*P*=0.046 and *P*=0.028, respectively). 391 differentially expressed genes and 8 signaling pathways were characterized 6 h after scraping; only 3 differentially expressed genes and 3 signaling pathways were screened out 2 d after treatment. The amounts of mRNAs or proteins for GLUT4, HK2, PFKM, PKM, LDHA (which belong to the GLUT4/glycolytic pathway), p-mTOR, p-4EBP1 (which belong to the AMPK/mTOR/4EBP1 pathway), and BDH1 were enhanced, and p-AMPK*α* was decreased after scraping therapy.

**Conclusions:**

Scraping therapy has therapeutic effects on rats with multifidus injury by promoting muscle regeneration via regulating GLUT4/glycolytic and AMPK/mTOR/4EBP1 signaling pathways.

## 1. Introduction

Nonspecific low back pain (NLBP) refers to acute and chronic pain in lumbosacral region localized between costal margin and inferior gluteal folds, with or without leg pain, excluding specific pathogenic factors [1, 2]. It accounts for 90% of cases in low back pain [1]. Pathological mechanism of NLBP is complex, which includes neurological, muscular, joint, and psychological factors. A study found that multifidus (MF) has an important role in maintaining spinal stability and performing normal physiological functions of the spine, and the damage and atrophy of MF were closely related to the development of NLBP [3]. MF is located deep in dorsal musculature, and it is attached to the spinous process, transverse process, and vertebral plates of the spine. Compared to other lumbar muscles, MF has larger cross-sectional areas and shorter fibers, which allow MF to generate tremendous forces in a small range of motion [4, 5].

There are many kinds of therapeutic applications towards NLBP including bed rest, pain medication, physical/rehabilitation therapy, and cognitive behavioral therapy [1, 2]. Nevertheless, each method has its own limitation or side effect. Bed rest is recommended as the primary treatment in some of the literature, but it is highly controversial, with some studies showing that bed rest is not only unbeneficial but may also have adverse effects compared to patients who maintain normal activities, especially when prolonged bed rest tends to cause atrophy of paravertebral muscles like MF, and induce complications including deep vein thrombosis [6–8]. Medications commonly used to treat NLBP include nonsteroidal anti-inflammatory drugs (NSAIDS), opioids, and antidepressants, all of which relieve pain symptoms through their respective targets of action but accompanied by inevitable side effects, with NSAIDS prone to gastrointestinal adverse effects and opioids predisposed to cause dizziness, nausea, and constipation [9, 10]. The limitation and addiction of these medications may compromise ultimate therapeutic effects. Physiotherapy, including heat therapy, electrotherapy, microwave, and other remedies, is now attracting more extensive clinical interest, as it is supposed to be capable of improving vascular microcirculation, promoting anti-inflammation, and relieving muscle spasm. However, its long-term efficacy on patients with low back pain is very limited [11]. Lumbar traction can relieve symptoms such as oozing, edema, and spasm of lumbar tissues and relax local muscles [12], but in a meta-analysis that analyzed 32 randomized controlled trials, researchers concluded that traction therapy was not significantly effective compared to the placebo group [13]. Exercise is currently one of the most recommended treatments for low back pain, and there are varieties of individualized projects aiming to relieve NLBP symptoms, restore daily function, and reduce recurrence rate by enhancing strength of patients' core spinal muscles, regulating physiological status, and improving psychological regulation [14, 15]. Exercise requires patients' long-term adherence, and some studies have pointed out that this therapy is not effective in relieving pain in the short term [16, 17].

Scraping therapy, also known as Gua Sha or coining therapy, is one of the characteristic external therapies in traditional Chinese medicine, which has been commonly practiced in Asia and partly distributed in Western countries. This modality involves scraping or rubbing lubricated area of the body repeatedly and unidirectionally with a smooth-edged instrument to intentionally raise transitory petechiae and ecchymosis which normally fade within 1 week [18]. Scraping therapy is empirically effective for acute or chronic pain and for other conditions such as respiratory diseases as well as musculoskeletal problems (from fibromyalgia to severe strain, spasm, or injury) [19]. However, most reports available mainly focused on therapeutic impacts as well as complications of scraping therapy [20], and there are only few studies that have limitedly investigated underlying mechanisms [21]. Implicit mechanism of therapeutic effects in scraping therapy makes its distribution confined to smaller regions compared with acupuncture, besides the noticeable abusive-like skin manifestations after treatment.

According to our long-term clinical observation on the effects of this modality, scraping therapy does relieve patients' muscular stiffness and soreness and partly improves muscle strength. Skeletal muscle has a strong regenerative capacity, which is closely related to the function of satellite cells (SCs) [22]. A growing amount of evidence suggested that SC function is largely dependent on two metabolic states of cells: oxidation and glycolysis [23]. Glycolysis plays an important role in muscle regeneration [24]. Adenosine 5′-monophosphate (AMP)-activated protein kinase (AMPK) is an important energy receptor in human body that is involved in a variety of signaling pathways and plays a regulatory role in glycometabolism, protein metabolism, lipid metabolism, and autophagy in the organism [25], thus also connected with the process of myocyte repair [26, 27]. In the present study, a rat model of lumbar multifidus injury was adopted to simulate NLBP of humans. We aimed to screen changed epigenetic expressions through transcriptomics after single scraping therapy and verify its myocyte-growth-promoting function, as well as the underlying mechanisms involved in glycolysis and AMPK pathway.

## 2. Materials and Methods

### 2.1. Animals

Male rats (Sprague–Dawley) were purchased from Charles River Laboratories (Beijing, China) at 6 to 7 weeks of age. They were kept under standardized pathogen-free conditions in the animal facility of 12 h light/dark cycles, temperature (24 ± 2°C), and 40%–50% relative humidity. All animal experiments have been approved by the Ethical Committee of Medicine of Navy Medical University (Shanghai, China; No. 2021GW0309) and have been conducted in accordance with the ethical standards laid down in the 1964 Declaration of Helsinki and its later amendments. A total of 54 rats were adaptively fed for 1 week and randomly divided into nine groups using a randomized digital table, namely, the blank group (K), the model 6h group (M6h), the model 1d group (M1d), the model 2d group (M2d), the model 3d group (M3d), the scraping 6h group (G6h), the scraping 1d group (G1d), the scraping 2d group (G2d), and the scraping 3d group (G3d), with 6 rats in each group, calculated by ANOVA using assumption that *α* = 0.05 and power = 0.80. All rats were euthanized by intraperitoneal overdose of 3% sodium pentobarbital (3 mL/kg) at appropriate time and the order of treatments and measurements were randomized. Apart from the conductor, no one was aware of the group allocation at the different stages of the experiment.

### 2.2. The Construction of Animal Model

We adopted the method described in the previous research [28]. The rats in the model and scraping groups were anesthetized by intraperitoneal injections of 3% sodium pentobarbital (1 mL/kg) and then fixed in a prone position with hair shaved. Muscle injury was induced by bilateral injections of 0.5% bupivacaine (BPVC; Sigma, USA) into MF at vertebral level L4 and L5. The injections of BPVC were made over 3 s with a 27-gauge needle, after which the needle was rotated out to facilitate the absorption of the drug. The needle was advanced into the muscle just beside the spinous process until it contacted the bone of vertebral arches. Then, the cannula was withdrawn 1 mm and the material deposited close to the bone to make sure that MF was injected. 100 *μ*L of BPVC was injected at each entry point and a total of 400 *μ*L were deposited. The whole operation was kept sterile.

### 2.3. The Manipulation of Scraping Therapy

**Figure 1:**
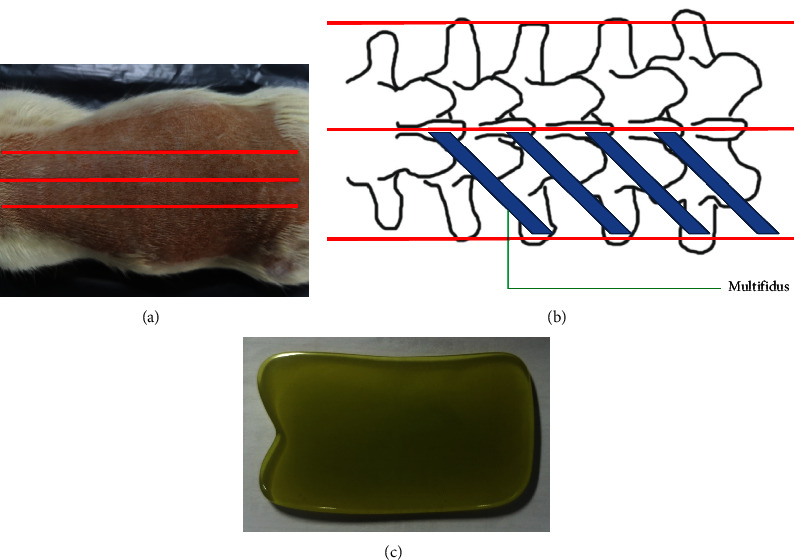
The areas treated and the instrument used by scraping therapy. (a) Three scraping lines were located on the middle of the back, and about 0.8 cm next to the midline on each side; the scraping length of each was equal. (b) The relative position among scraping lines, spine, and multifidus. (c) The instrument used to scrape is made of bull horn (this is an original figure created by the authors).

The rats in scraping groups were given scraping therapy and the target range was from the first thoracic vertebra down to the sacrum on the back (three scraping lines were located on the middle of the back, and about 0.8 cm next to the midline on each side; the scraping length of each was equal, Figure 1(a)). The selected skin area was shaved and wiped with glycerin for lubrication. The rats were then scraped 30–35 times for each scraping line with a smooth-edged instrument made of bull horn (Figure 1(c)) in a unidirectional manner. The scraping force was optimized and standardized at 7-8 N; the frequency was 40 times/min, and the total time of therapy was about 3 min.

### 2.4. Skin Temperature Measurement

Skin temperatures at the vertebral L4, L5, and L6 were measured at approximately 10 cm above the skin of the rat's back using an infrared thermal imager (FLIR, USA). Rats were tested for the baseline temperature before scraping therapy, and those in the scraping groups were additionally tested both at the time immediately after scraping and before harvested.

### 2.5. The Assessment of Tactile Allodynia

The assessment of tactile allodynia was performed as previously described [29]. Testing was performed during the day portion of the circadian cycle only (06:00–18:00 h). Rats were placed in a von Frey testing cage with a wire mesh bottom which allowed full access to the paws. Behavioral accommodation was allowed for approximately 20 min, until cage exploration and major grooming activities ceased. The area tested was the mid-plantar hind paws, in the sciatic nerve distribution, avoiding the less sensitive tori (footpads). The paw was touched with 1 of a series of 8 von Frey hairs with logarithmically incremental stiffness (0.41, 0.70, 1.20, 2.00, 3.63, 5.50, 8.50, and 15.10 g) (NC12775-99 Touch-Test, USA). The von Frey hair was presented perpendicular to the plantar surface with sufficient force to cause slight buckling against the paw, and it was held for approximately 6–8 s. Stimuli were presented at intervals of 15 s, allowing for apparent resolution of any behavioral responses to previous stimuli. A positive response was recorded if the paw was sharply withdrawn or the rat flinched immediately upon removal of the hair. Ambulation was considered an ambiguous response, and in such cases, the stimulus was repeated. The 50% withdrawal threshold was determined using the up-down method through the formula: 50% g threshold = (10^[*X*_*f*_+*kδ*]^)/10,000 [30]. Rats were tested for the baseline pain thresholds, and those in the model and scraping groups were tested 24 h after BPVC injection and at the time before sacrificed, and each rat was tested in both hind paws.

### 2.6. Immunohistochemistry

The skin and MF which had been harvested were fixed in 4% paraformaldehyde for 48 h and then washed with running water to remove residual paraformaldehyde and impurities. The tissues were sealed and dehydrated in gradient concentrations of ethanol (50%, 70%, 85%, 95%, and anhydrous ethanol) for 2 h each. The washed and dehydrated tissues were soaked in a mixture of anhydrous ethanol and xylene for 2 h followed by xylene immersion for 2 h. The process was repeated once to make the tissues transparent. The tissues were first impregnated in a mixture of melted paraffin and xylene in equal amounts for 2 h, and then placed in paraffin for 2 h twice. The transparent tissue blocks were placed in the melted paraffin wax and kept warm in the molten wax chamber. The blocks were sliced at 5 *μ*m using a microtome (RM2235, Leica, Germany). The tissue slices were put into an incubator (Huitai Company, China) at 65°C for baking for 30 min. The slices were then put into xylene for 15 min twice, and dehydrated in gradient concentrations of ethanol (100%, 95%, 90%, 80%, and 70% ethanol to water), each for 15 min. The tissue sections were put into hematoxylin solution, stained for 5 min, rinsed under running water for 3 min; 1% hydrochloric acid alcohol fractionation for 2-3 s, rinsed under running water for 15 min, and then put into 95% ethanol for 30 s of dehydration; the tissue sections were then put into alcohol eosin solution, stained for 2 min, and put into 95% ethanol for 3 min twice, and anhydrous ethanol for 3 min twice. The slices were transparentized with xylene for 3 min twice, blocked with neutral resin, dried at 65°C, and were observed using a microscope (DMi8, Leica, Germany). Cross-sectional area (CSA) of MF fiber (primary outcome) was analyzed by randomly selecting 30 fibers using ImageJ analysis software (National Institutes of Health, https://rsb.info.nih.gov/ij/).

### 2.7. mRNA-seq and Data Analysis

Total RNAs from MF tissues were extracted using TRIzol reagent (Life Technologies, USA) according to the manufacturer's instructions. RNA purity was measured using NanoDrop™ One/OneC (ThermoFisher Scientific, USA) and was quantified with Qubit™ RNA HS Assay Kit (Qiagen, German). RNA integrity (RIN) was analyzed using Agilent 4200 TapeStation System. The mRNA with polyA structure was captured using magnetic beads with Oligo dT. Then, the fragmented mRNA was used as template and random oligonucleotides used as primers. The first strand of cDNA was synthesized in the M-MuLV reverse transcriptase system, and dNTPs degraded from RNA with RNaseH were used in the DNA polymerase I system to synthesize the second strand of cDNA [31]. After purified and repaired, cDNA of about 200 bp was screened with AMPure XP beads and amplified through PCR to construct the library, which was sequenced on an Illumina HiSeq PE150 sequencer [32]. Sequenced fragments were mapped to reference genome with HISAT2 software [33]. Gene expression analysis was performed using HTSeq and value conversion was reported in fragments per kilobase per million (FPKM).

### 2.8. Real-Time Reverse Transcription-Quantitative Polymerase Chain Reaction

Snap-frozen MF tissues were rapidly homogenized and RNA was isolated by using RNeasy mini kit (Qiagen, German). The transcription level of the isolated RNA was quantified by Mutiskan™ GO (ThermoFisher Scientific, USA). Total RNA was reverse transcribed to cDNA by First Strand cDNA Synthesis Kit (Invitrogen, USA). RT-PCR was implemented using Roche480II Real-Time PCR System (Roche Diagnostics, China) with QuantiNova SYBR Green PCR Kit (Qiagen, German). The quantification of genes of interest was normalized to a reference gene glyceraldehyde-3-phosphate dehydrogenase (Gapdh) and expressed as a relative fold-change of the Hexokinase 2 (Hk2) in the K group by a standard 2^−ΔΔCT^ method [34, 35]. The following primers (Genewiz, Suzhou, China) were used in this study: Gapdh (NM_017008.4), Hk2 (NM_012735.2), Pfkm (NM_031715.1), Slc2a4 (NM_012751.1), Prkaa1 (NM_019142.3), and Bdh1 (NM_053995.3).

### 2.9. Western Blot Analysis

Western blot analysis was performed as described [36]. Equal amounts of protein (15–20 *μ*g) were separated by sodium dodecyl sulfate-polyacrylamide gel electrophoresis and transferred onto polyvinylidene difluoride membrane using Criterion XT Precast gels (Bio-Rad, USA). Membranes were blocked with 5% BSA for 2 h; then the corresponding antibodies GLUT4 (1 : 1000, #347063, Zen Bio), HK2 (1 : 1000, #200569, Zen Bio), PFKM (1 : 1000, #ab154804, Abcam), PKM (1 : 1000, #200667-1A7, Zen Bio), LDHA (1 : 500, #384822, Zen Bio), AMPK*α* (1 : 1000, #ab207442, Abcam), p-AMPK*α* (1 : 1000, #AP0116, ABclonal), mTOR (1 : 1000, #380411, Zen Bio), p-mTOR (1 : 1000, #5536, Cell signalling), 4EBP1 (1 : 500, #306002, Zen Bio), p-4EBP1 (1 : 500, #ab278686, Abcam), and Actin (1 : 2000, #200068-8F10, Zen Bio) were incubated overnight at 4°C; finally, blots were incubated with secondary antibodies at room temperature for 2 h. Bands were visualized with the gel imaging system (Syngene, UK) and analyzed with ImageJ analysis software (National Institutes of Health, https://rsb.info.nih.gov/ij/). The research met all the requirements of ARRIVE checklist and most of the requirements of CONSORT checklist if applicable (see supplemental files, named S1 ARRIVE Checklist and S2 CONSORT 2010 Checklist).

### 2.10. Pilot Study

A total of 9 rats were adaptively fed for 1 week and randomly divided into 3 groups, namely, the blank group, the model 1d group (M1d), and the scraping 1d group (G1d), with 3 rats in each group. The results of cross-sectional area for each group are as follows in Table 1.

### 2.11. Statistics

All statistical analyses were performed using SPSS 23.0 software (all the raw data were included in the supplemental file, named S3 raw data). Quantitative data were reported as the mean ± standard error. One-way analysis of variance (ANOVA) followed by Fisher's least significant difference (LSD) post hoc test was performed for multiple comparisons of cross-sectional area, and expressions of mRNA and proteins, and paired *t*-test was performed for data of skin temperature and allodynia. Shapiro–Wilk test was used for the normality assessment. Statistics of tactile allodynia were not normally distributed, and Wilcoxon signed-rank test was used. Other data were all normally distributed. For the comparison of skin temperature and allodynia, the independent variables were time intervals after scraping therapy in the scraping groups and time intervals without scraping therapy in the blank group and the modeling groups; the dependent variables were skin temperature and tactile allodynia, respectively. For the comparison of cross-sectional area and expressions of mRNA and proteins, the independent variable was whether given scraping therapy or not; the dependent variables were cross-sectional area and expressions of mRNA and proteins, respectively. The mathematical formula in use for the sample size calculation was *n* =[((*μ*_*α*_+*μ*_*β*_)*σ*/*δ*)]^2^+(1/4)*μ*_*α*_^2^, and we used the program called “Tests for Two Means” of PASS 11 for calculating. *σ* is the standard deviation of the change in cross-sectional area of lumbar multifidus of rats after scraping therapy, *δ* is the difference value in the population mean of the change in cross-sectional area of lumbar multifidus of rats, and *μ*_*α*_ and *μ*_*β*_ are values of *μ* corresponding to *α* and *β*, respectively, which had all come from our preliminary experiments. For all analyses, a *p* value of <0.05 was considered to be statistically significant.

## 3. Results

### 3.1. Scraping Therapy Raised Transitory Petechiae and Ecchymosis Which Would Fade in about Three Days and Promoted the Recovery of Myocytes

**Figure 2:**
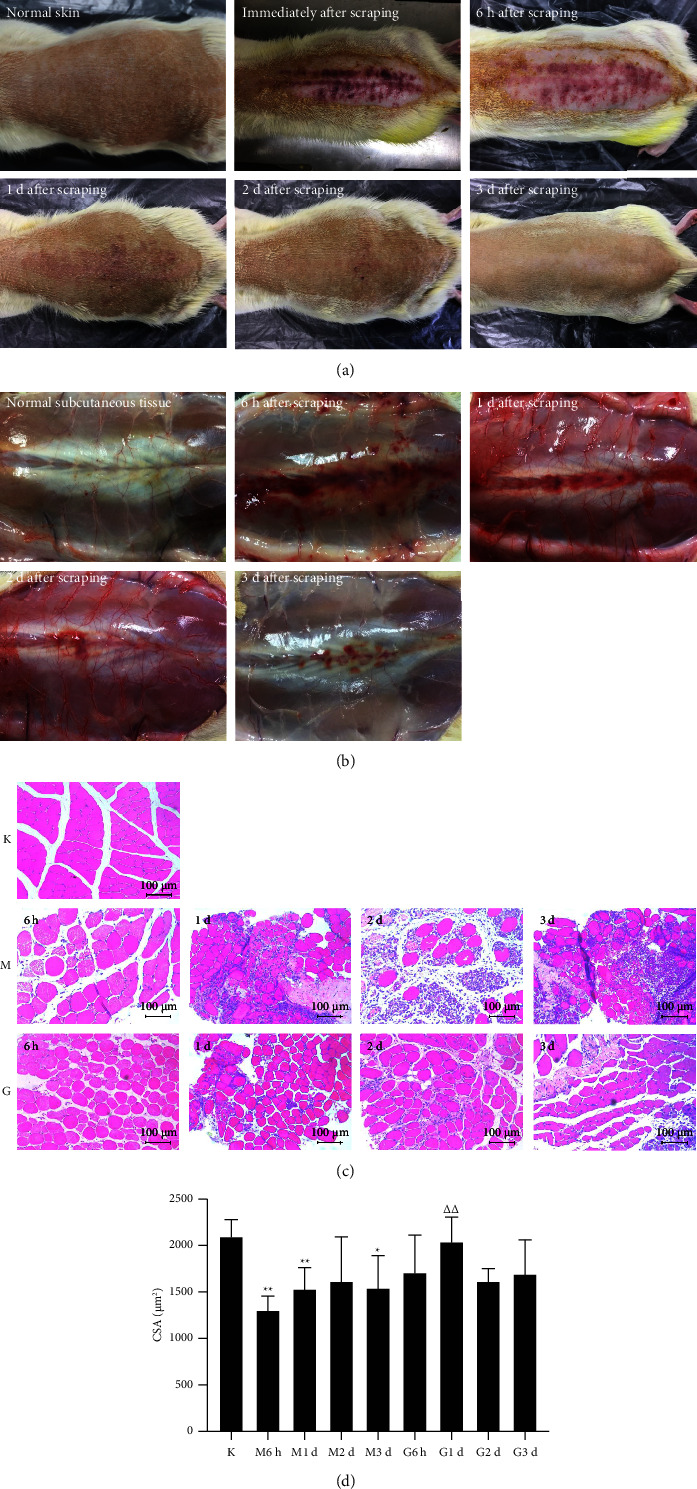
The effects of scraping therapy on rats' tissues. Changes on skin (a) and subcutaneous tissues (b) at different time points after scraping therapy gradually recovered in about 3 days. (c, d) Representative hematoxylin and eosin images and quantitative data for CSA of MF. K for the blank group, M for the model group, and G for the scraping group. ^*∗*^*P* < 0.05 versus K, ^*∗∗*^*P* < 0.01 versus K, and △△*P* < 0.01 versus M at the same time. *N* = 6 per group. Scale bars: 100 *µ*m (this is an original figure created by the authors).

Transitory petechiae and ecchymosis both on and beneath rats' skin raised by scraping therapy gradually faded in about 3 days (Figures 2(a) and 2(b)). 3 days after scraping, skin surface of rats basically restored to the baseline level, with no visible ecchymosis, and subfascial stasis was basically absorbed, with a few scattered light red petechiae in sight. There were visibly less cell debris, fewer inflammatory cells, and more neonatal myocytes in the G groups compared with the M groups at the same time (Figure 2(c)). CSA of MF was significantly smaller 30 h, 2 d, and 4 d after modeling (*P*=0.007, *P*=0.001, and *P*=0.015, respectively) and was significantly larger in the G1d group than in the M1d group 1 d after scraping (*P*=0.002) (Figure 2(d) and Table 2). However, there were no significant differences in CSA between the M group and the G group at other time points.

### 3.2. Scraping Therapy Increased Rats' Skin Temperature Transiently and Partly Enhanced Withdrawal Threshold of Rats' Hindlimb

**Figure 3:**
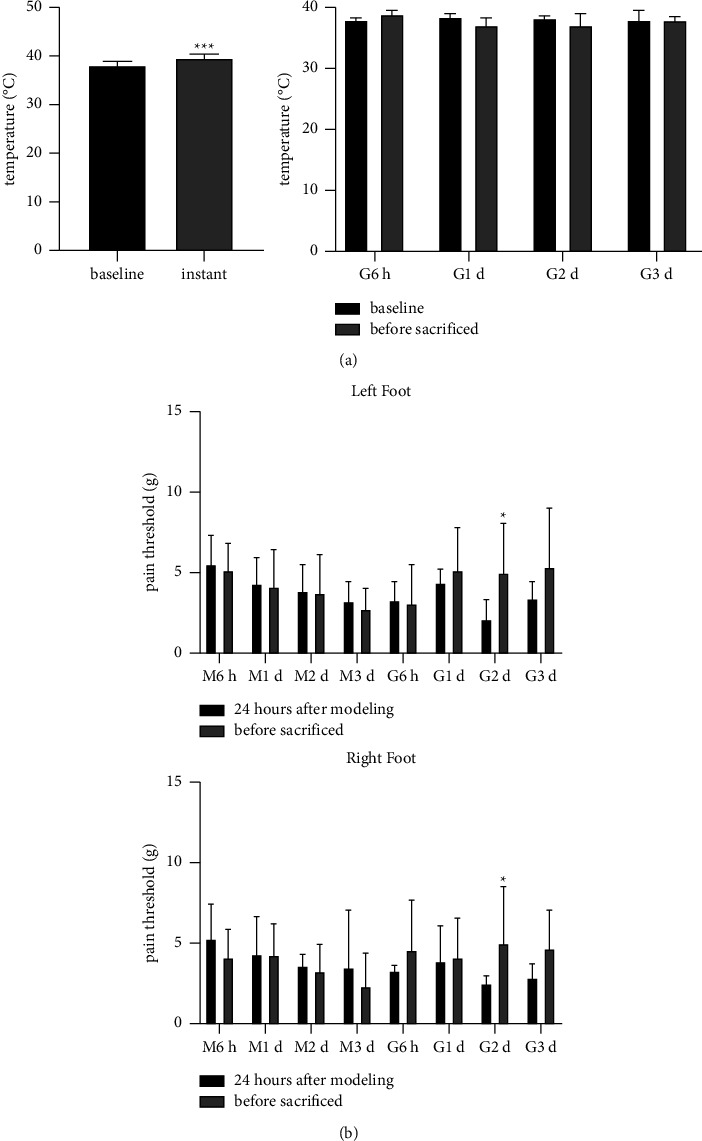
Changes on skin temperature and allodynia of rats after scraping therapy. (a) Scraping therapy increased local skin temperature immediately after manipulation. ^*∗∗∗*^*P* < 0.001, *n* = 24. No significant differences were identified at other time points after scraping. *N* = 6 per group. (b) Changes on pain threshold of both hind paws were observed. ^*∗*^*P* < 0.05, *n* = 6 per group.

Local skin temperature significantly increased immediately after scraping (*P* < 0.001), while no significant differences were found at other time points (Figure 3(a) and Table 3). Pain threshold (also called withdrawal threshold) increased in the 2nd day after scraping (*P*=0.046 and *P*=0.028, respectively) with no prominent differences recorded at other time points (Figure 3(b), Tables 4 and 5). Our previous findings indicated that single scraping treatment may have limited effect of pain relieving, which had little to do with temperature increase of local tissues.

### 3.3. The Changes of mRNA Expressions Induced by Scraping Therapy Were Analyzed through mRNA Sequencing

**Figure 4:**
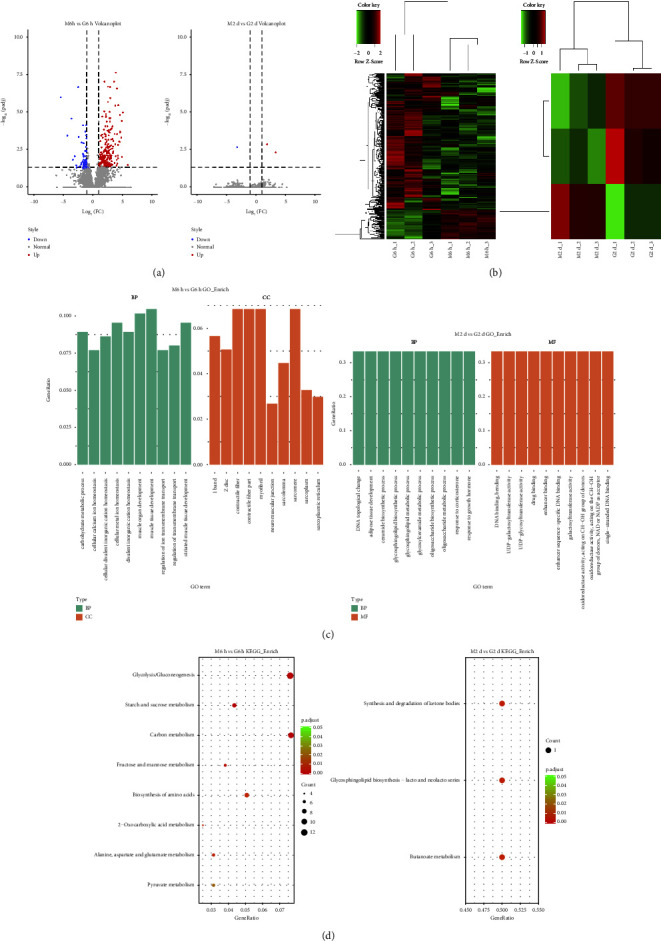
The results of mRNA sequencing. Volcano plots (a) and cluster plots (b) showed differentially expressed genes between the M6h group and the G6h group as well as between the M2d group and the G2d group. Red color represented upregulated genes and green color denoted downregulated genes. (c, d) Functional enrichment results in GO and KEGG. Dot size indicated the number of differentially expressed genes enriched in specific pathways and redder color denoted more significance. *N* = 3 per group.

There were 391 differentially expressed genes in the G6h group compared with the M6h group, including 322 upregulated genes and 69 downregulated genes. Only 3 differentially expressed genes were identified between the G2d group and the M2d group, with 2 upregulated and 1 downregulated genes (Figures 4(a) and 4(b)). 2 upregulated genes were *Hmgb2l1* and *Bdh1*, and 1 downregulated gene was *B3galt2*.

The results of gene ontology (GO) enrichment analysis of differentially expressed genes between the G6h group and the M6h group included the following: (i) biological processes: mainly enriched in skeletal muscle development, carbohydrate catabolism, and intracellular metal ion homeostasis; (ii) cellular composition: mainly enriched in muscle nodes, contractile fiber fraction, and myogenic fibers; and (iii) molecular functions: no relevant functions were enriched. The results between the G2d group and the M2d group included the following: (i) biological processes: mainly enriched in sphingolipid metabolism, oligosaccharide metabolism, and response to growth hormone; (ii) molecular functions: mainly enriched in DNA binding, galactosyltransferase activity, and enhancer binding; and (iii) cellular composition: no relevant functions were enriched (Figure 4(c)).

The main pathways with a significant concentration of differentially expressed genes between the G6h group and the M6h group were analyzed through Kyoto Encyclopedia of Genes and Genomes (KEGG) enrichment, and eight signaling pathways, including glycolysis/glycogen isomerization, starch and sucrose metabolism, carbon metabolism, and fructose and mannose metabolism, were obtained. The main different pathways between the G2d group and the M2d group were three signaling pathways, namely, ketone body synthesis and degradation, methyl butyrate metabolism, and sphingolipid biosynthesis (Figure 4(d)).

### 3.4. Scraping Therapy Altered the Expression of Transcription Factors Functioning in Glycolysis and Ketone Body Metabolism

**Figure 5:**
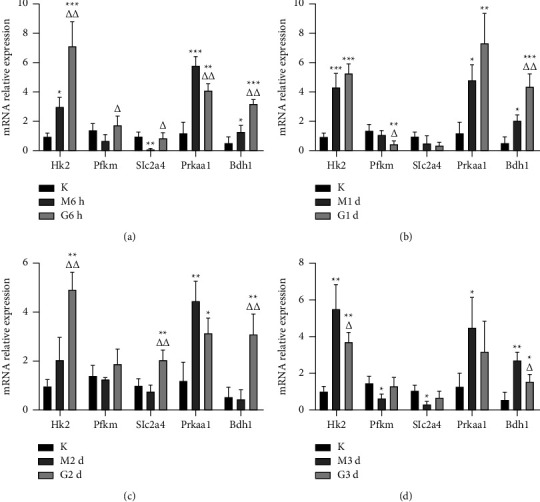
The effects of scraping therapy on targeted gene expression in MF at different time points after manipulation. Quantitative analysis of RT-PCR for the levels of *Hk2*, *Pfkm*, *Slc2a4*, *Prkaa1*, and *Bdh1* mRNA expressions 6 hours (a), 1 day (b), 2 days (c), and 3 days (d) after scraping therapy. K for the blank group, M for the model group, and G for the scraping group. ^*∗*^*P* < 0.05, ^*∗∗*^*P* < 0.01, and ^*∗∗∗*^*P* < 0.001 versus K; △*P* < 0.05 and △△*P* < 0.01 versus M at the same time. *N* = 3 per group.

We next sought to verify the results of mRNA-seq and identify molecular mechanisms for the increase in CSA of MF with scraping therapy by analyzing relevant transcription factors of anabolic and catabolic pathways. As shown in Figures 5(a)–5(d), mRNA analysis of genes associated with glycolysis and ketone body metabolism pathways showed that expressions of *Hk2* (*P*=0.002, *P*=0.112, and *P*=0.003, respectively) (which is the first rate-limiting enzyme in glycolysis) and *Bdh1* (*P* < 0.001, *P*=0.003, and *P* < 0.001, respectively) (which promotes synthesis of ketone body) were increased in 2 days after treatment. Other gene expression levels of *Pfkm*, *Slc2a4*, and *Prkaa1* fluctuated during the whole experiment period, which may have some underlying mechanisms to be uncovered, and we wanted to conduct a preliminary discussion in the next section. The statistics for mRNA expressions are as follows in Table 6.

### 3.5. Scraping Therapy Advanced the Expression or Phosphorylation of Proteins Involved in Glucose Transport, Glycolysis, and AMPK/mTOR/4EBP1 Pathways Six Hours after Treatment

**Figure 6:**
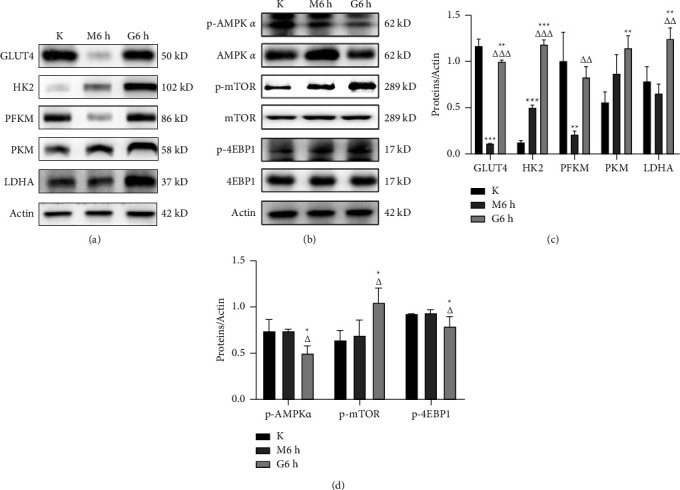
The effects of scraping therapy on targeted protein expression or phosphorylation in MF 6 hours after manipulation. (a, b) Representative immunoblots showed the levels of targeted protein expression or phosphorylation in MF. (c, d) Quantitative analysis of Western blots for the levels of GLUT4, HK2, PFKM, PKM, LDHA, p-AMPK*α*, p-mTOR, and p-4EBP1 proteins in the three groups. The expression level of each targeted protein was normalized with a Western blot antibody to actin. ^*∗*^*P* < 0.05, ^*∗∗*^*P* < 0.01, and ^*∗∗∗*^*P* < 0.001 versus K; △*P* < 0.05, △△*P* < 0.01, and △△△*P* < 0.001 versus M. *N* = 3 per group.

Since differentially expressed genes had been recorded, we then further analyzed the proteins involved in the pathways mentioned previously from the extractions of MF 6 hours after scraping through Western blot analysis. As indicated in Figures 6(a)–6(d), compared with the M group, scraping therapy resulted in an increased level of GLUT4 (*P* < 0.001), and it also led to increased expressions of HK2 and PFKM (*P* < 0.001 and *P*=0.006, respectively), and a trend for increased PKM expression (*P*=0.066) (which are three rate-limiting enzymes in glycolysis), as well as increased LDHA expression (*P*=0.001) (which is the enzyme for the final step of glycolysis). In addition, this manipulation inhibited the phosphorylation of AMPK*α* (*P*=0.012) and enhanced the phosphorylation of mTOR and 4EBP1 (*P*=0.021 and *P*=0.035, respectively). Taken together, scraping therapy can rapidly enhance glycolysis and regulate the AMPK/mTOR/4EBP1 pathway. The statistics for protein expressions are as follows in Table 7.

## 4. Discussion

Scraping therapy, termed Gua Sha, which is an effective treatment modality of traditional Chinese medicine, has a wide range of applications and can treat more than 30 kinds of diseases associated with the nervous system, motor system, respiratory system, digestive system, cardiovascular system, and endocrine system. In this study, we observed the effect of scraping therapy on pathological changes of MF cells. Within 3 days after treatment, compared with the model groups, the disruption of MF cells in the scraping groups was alleviated; the infiltration of inflammatory factors was decreased, and the CSA of MF had an increase tendency, indicating that repair process of myocytes was accelerated. Skin temperature of rats after scraping increased immediately compared with the baseline, from which we can presumed that scraping therapy can temporarily increase skin surface temperature, promote local tissue microcirculation and energy metabolism, and regulate the microenvironment of the body in a short period, which is consistent with the findings of Xu et al. [37]. Withdrawal threshold of rats' hind paws in the scraping group was elevated on the 2nd day after intervention compared with the model group, indirectly demonstrating that scraping therapy could alleviate symptoms of low back pain to some extent, and the effect may be related to the enhancement of local microcirculation and other unelaborated mechanisms of action [19].

Skeletal muscle has a strong regenerative capacity and can rapidly restore muscle strength even in the presence of extensive muscle fiber necrosis, which is closely related to the function of satellite cells (SCs), a class of skeletal muscle stem cells with the ability to promote muscle growth and tissue repair, located in the niche between the myofibril and basement membrane on the surface of the muscle fiber [22, 38]. Under normal conditions, SCs are in mitotic quiescence (G0 phase), and once the microenvironment in which SCs are located changes, such as when muscle tissue injury occurs, they exit G0 phase and instead begin to proliferate rapidly and migrate to the targeted site, fusing with damaged myogenic fibers to regenerate and restore their function [38–40]. In addition to transcriptional regulation, SC metabolic regulation is another key regulatory mechanism. A growing amount of evidence suggested that SC function is largely dependent on two metabolic states of the cells: oxidation and glycolysis [23]. Glycolysis is the process of conversion from one molecule of glucose to two molecules of pyruvate along with two molecules of adenosine triphosphate (ATP) in the presence of multiple enzymes. While in an oxygen-rich environment, cells can use oxidative phosphorylation (OXPHOS) to produce ATP more efficiently by oxidizing pyruvate to acetyl-coenzyme A (acetyl-CoA) followed by productions of carbon dioxide and water through the tricarboxylic acid cycle (TCA cycle) that occurs in the mitochondria, producing an average of 34 molecules of ATP per molecule of glucose. In contrast, glycolysis seems to be an inefficient energy-producing metabolic pathway, but it can play an important role in rapid provision of ATP and the synthesis of key cellular macromolecules [41–43]. Ryall et al. [24] suggested that skeletal muscle stem cells shift their metabolic processes from original fatty acid oxidation to a glycolytic-based metabolic pattern during the transition from quiescence to proliferative phase. Chen et al. [44] used a YY1-knockdown mouse model to investigate how SC uses glycolytic metabolism to activate its own proliferative and differentiation functions. The researchers found that muscle regeneration was severely reduced after acute injury in mice, and SC showed defective autonomic activation and proliferative capacity. Transcriptome analysis revealed that YY1 could inhibit mitochondrial gene expression and activate Hif1*α*-mediated glycolytic gene expression, whereas YY1 gene deletion leads to upregulation of mitochondrial gene expression and inhibition of glycolysis, resulting in defective SC activation and proliferative capacity, demonstrating that YY1 can play a key regulatory role in SC metabolic reprogramming by regulating both mitochondrial and glycolytic pathways.

Glucose is transported into cells in two main ways: one is by energy-consuming counter-concentration gradient cotransport with Na^+^, which occurs mainly in renal tubular epithelial cells, and small intestinal mucosal cells; the other way is through glucose transporter proteins on cell membrane, which is a non-energy-consumingcis-concentration gradient transport process [45]. There are five isoforms of glucose transporter proteins, of which facilitated glucose transporter member 4 (GLUT4), encoded by *solute carrier family 2 member 4* gene (*Slc2a4*), is mainly found in adipose and muscle tissues and encodes glucose transport process, which is a rate-limiting step in glucose metabolism by skeletal muscle cells using glucose [46]. Hexokinase (HK) is the rate-limiting enzyme of glycolysis, which catalyzes the production of glucose 6-phosphate (G-6-P) from glucose and this reaction is irreversible. HK has 4 subtypes, among which hexokinase 2 (HK2) is mainly present in skeletal muscle cells and is a key enzyme promoting glycolysis of skeletal muscle cells [47]. Fructokinase 6-phosphofructokinase 1 (PFK1) catalyzes the production of fructose 1,6-diphosphate from fructose 6-phosphate, which is the second rate-limiting enzyme in the glycolytic pathway. There are three isozymes, and we analyzed PFKM (phosphofructokinase, muscle) in our study because the specimens used were MF tissues. The third rate-limiting enzyme in the glycolytic pathway, pyruvate kinase (PK), catalyzes the phosphoenolated pyruvate to generate ATP and consists of M-type and L-type isoenzyme. PKM was the one we tested as it is mainly distributed in skeletal and cardiac muscles. In the current experiment, gene expression levels of *Hk2*, *Pfkm*, and *Slc2a4* increased within 2 days except for temporary decline of the latter two gene expression on the 1st day after scraping therapy, indicating that glycolysis was generally enhanced within 2 days after treatment, especially 6 hours after manipulation. This is consistent with the significant increase of protein expression levels of GLUT4, HK2, PFKM, PKM, and LDHA which are all involved in glycolysis.

AMPK is composed of the catalytic *α* subunit and the regulatory *β* and *γ* subunits, and the *α* subunit has two isoforms, *α*1 and *α*2. AMPK*α*1 is widely present in different tissues and is encoded by the *Prkaa1* gene [48]. Fu et al. [49] found that AMPK*α*1 has an important role in muscle regenerative capacity, which can activate SC aerobic glycolysis through nonclassical sonic hedgehog (Shh) signaling pathway, thus promoting SC activation and proliferation. Mammalian target of rapamycin (mTOR) likewise plays an important role in life activities of organism, participating in various metabolic processes such as protein translation, lipid synthesis, inhibition of autophagy, and cell cycle regulation [50], which can be inhibited by AMPK. Eukaryotic translation initiation factor 4E (eIF4E)-binding protein 1 (4EBP1) is one of the important target proteins downstream of mTOR, which is phosphorylated and dissociated from eIF4E. The free eIF4E exposes active binding sites and binds to other translation initiation factors, thus promoting the translation of mRNA and increasing protein synthesis, which is an indispensable and critical component of cell proliferation [51]. In our study, *Prkaa1* gene expression decreased within 3 days compared with the model groups, except for the temporary increase on the 1st day after scraping therapy, implying that the AMPK activity was relatively inhibited, especially 6 hours after treatment. Western blot results showed that 6 hours after scraping, compared with the model group, the AMPK phosphorylation level decreased and mTOR and 4EBP1 phosphorylation levels increased in the scraping group. The regulation of this signaling pathway was beneficial to protein synthesis and skeletal muscle regeneration. Taken together, the AMPK/mTOR/4EBP1 pathway was regulated by scraping therapy in a short period. While AMPK can activate glycolytic pathway, when AMPK activity is inhibited, myocytes are prevented from appearing to overproliferate similar to growing mode of cancer cells due to excessive elevation of the glycolytic activity.

Ketone bodies are small molecules synthesized in the liver primarily from fat in the presence of fasting, prolonged exercise, or inadequate carbohydrate intake [52]. They circulate in the blood, with the ability to cross blood-brain barrier, and are taken up by energy-demanding peripheral tissues, where they are oxidized in mitochondria to produce acetyl-CoA, which drives the regeneration of ATP [53]. The two major endogenous ketone bodies are acetoacetate (AcAc) and *β*-hydroxybutyrate (BHB), respectively, with BHB more abundant and stable in blood. In the presence of catalytic BDH1 (D-*β*-hydroxybutyrate dehydrogenase, mitochondrial), AcAc is converted to BHB in the liver and the reaction is reversed in peripheral tissues, accounting for the final step in ketone synthesis in the liver and the first step in ketone catabolism in peripheral tissues, respectively [54]. Molecular functions of ketone bodies include providing energy, reducing inflammation and oxidative stress, promoting muscle regeneration, and participating in lipid metabolism. AcAc has multiple signaling activities distinct from BHB, including binding to GPR43 to regulate lipid metabolism [55] and the activation of MEK1-ERK1/2 cell cycle protein D1 signaling pathway to accelerate muscle cell proliferation and muscle regeneration [56]. In skeletal muscle tissue, a rise in *Bdh1* expression is associated with an increase in the conversion of BHB to AcAc and an increase in ketone body catabolic energy supply. *Bdh1* gene expression was significantly enhanced within 2 days after scraping and decreased on the 3rd day, indicating that the modality promoted energy supply produced by ketone metabolism, which however need to be further verified.

## 5. Conclusions

**Figure 7:**
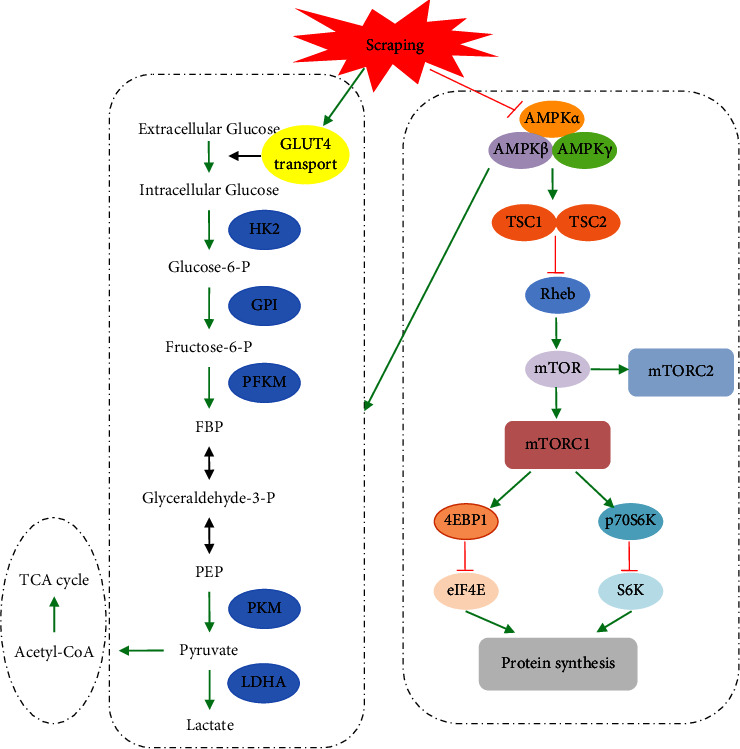
Scraping therapy-regulated GLUT4/glycolytic and the AMPK/mTOR/4EBP1 signaling pathways. After scraping therapy, genes and proteins involved in glycolysis such as GLUT4, HK2, PFKM, and PKM were all upregulated; AMPK*α* was downregulated and p-mTOR and p-4EBP1 were upregulated, thus promoting protein synthesis.

In summary, scraping therapy showed a clear therapeutic effect on injured multifidus and could alleviate pain symptoms to some extent in rats. The potential mechanisms of its efficacy that we demonstrated were as follows: (i) regulating the GLUT4/glycolytic pathway and activating myosatellite cell proliferation and (ii) regulating the AMPK/mTOR/4EBP1 signaling pathway and promoting protein synthesis (Figure 7). At a later stage, *in vivo* experiments including gene knockdown and overexpression techniques need to be used to further validate the role of GLUT4/glycolytic and AMPK/mTOR/4EBP1 signaling pathways in promoting myocytes repair and regeneration, as well as regulatory mode of scraping therapy.

## Figures and Tables

**Table 1:** Results of cross-sectional area of rats' lumbar multifidus fiber (*µ*m^2^).

Groups	CSA (mean ± SD, *μ*m^2^)	*P*
Blank	2090.76 ± 132.83	—
M1d	1585.51 ± 93.74	<0.001
G1d	2000.33 ± 58.95	

**Table 2:** Statistics for cross-sectional area of rats' lumbar multifidus fiber (*µ*m^2^).

	Sample size	Mean ± SD	95% CI for mean		Minimum	Maximum	*P* (vs. rK)	*P* (vs. M)
			Lower limit	Upper limit				
K	6	2100.05 ± 188.24	1902.50	2297.59	1833.05	2307.68	—	—
M6h	6	1300.61 ± 157.80	1135.01	1466.21	1088.39	1568.19	0.007	—
M1d	6	1526.74 ± 246.53	1268.03	1785.45	1307.14	1946.85	0.001	—
M2d	6	1630.49 ± 477.70	1129.18	2131.81	1227.80	2400.42	0.069	—
M3d	6	1537.87 ± 353.72	1166.66	1909.07	1067.95	2028.83	0.015	—
G6h	6	1715.52 ± 409.20	1286.09	2144.95	1179.66	2103.44	0.281	0.529
G1d	6	2037.55 ± 275.98	1747.92	2327.17	1697.55	2444.86	0.658	0.002
G2d	6	1619.59 ± 148.65	1463.60	1775.58	1418.99	1863.33	0.105	1.000
G3d	6	1684.48 ± 382.70	1207.33	2176.52	1265.50	2407.15	0.064	0.461

**Table 3:** Statistics for rats' skin temperature (°C).

		Sample size	Mean ± SD	95% CI for mean		Minimum	Maximum	*P*
				Lower limit	Upper limit			
G	Baseline	24	38.05 ± 0.93	37.66	38.44	36.40	40.00	<0.001
	Instant		39.33 ± 0.76	39.00	39.65	38.00	41.20	

G6h	Baseline	6	37.87 ± 0.58	37.26	38.47	37.40	38.70	0.050
	6h		38.87 ± 0.73	38.10	39.63	37.90	40.00	

G1d	Baseline	6	38.13 ± 0.73	37.37	38.90	37.30	38.90	0.239
	1d		37.13 ± 1.19	35.88	38.38	35.10	38.20	

G2d	Baseline	6	38.25 ± 0.64	37.58	38.92	37.40	38.90	0.348
	2d		37.32 ± 1.67	35.57	39.07	35.20	39.00	

G3d	Baseline	6	37.95 ± 1.61	36.26	39.64	36.40	40.00	0.876
	3d		37.78 ± 0.93	36.81	38.76	36.40	38.80	

**Table 4:** Statistics for rats' tactile allodynia (g) (left hindlimb).

		Sample size	Mean ± SD	95% CI for mean		Minimum	Maximum	*P*
				Lower limit	Upper limit			
M6h	Baseline	6	5.55 ± 1.79	3.67	7.42	3.43	7.86	0.068
	6h		5.08 ± 1.67	3.33	6.83	3.09	7.30	

M1d	Baseline	6	4.37 ± 1.62	2.67	6.07	2.56	7.30	0.465
	1d		4.09 ± 2.35	1.62	6.55	1.19	7.30	

M2d	Baseline	6	3.89 ± 1.51	2.30	5.47	2.56	6.03	0.917
	2d		3.76 ± 2.32	1.32	6.20	0.96	6.98	

M3d	Baseline	6	3.24 ± 1.26	1.92	4.56	1.81	4.85	0.225
	3d		2.69 ± 1.33	1.29	4.09	1.19	4.73	

G6h	Baseline	6	3.30 ± 1.16	0.69	10.72	2.15	11.86	0.600
	6h		3.05 ± 2.48	0.45	5.65	0.96	6.12	

G1d	Baseline	6	4.30 ± 0.93	3.32	5.28	3.09	5.57	0.249
	1d		5.11 ± 2.63	2.35	7.86	1.92	7.79	

G2d	Baseline	6	2.14 ± 1.14	0.75	5.62	1.15	7.43	0.046
	2d		4.93 ± 3.14	1.64	8.23	1.92	9.74	

G3d	Baseline	6	3.28 ± 1.25	1.96	4.59	1.53	4.73	0.345
	3d		5.36 ± 3.61	1.57	9.15	2.60	11.45	

**Table 5:** Statistics for rats' tactile allodynia (g) (right hindlimb).

		Sample size	Mean ± SD	95% CI for mean		Minimum	Maximum	*P*
				Lower limit	Upper limit			
M6h	Baseline	6	5.24 ± 2.10	3.03	7.44	2.18	7.86	0.225
	6h		4.05 ± 1.74	2.23	5.87	2.18	6.12	

M1d	Baseline	6	4.33 ± 2.27	1.95	6.72	1.15	7.43	0.893
	1d		4.26 ± 1.93	2.23	6.28	1.81	6.55	

M2d	Baseline	6	3.53 ± 0.72	2.77	4.28	2.60	4.54	0.686
	2d		3.18 ± 1.82	1.27	5.08	1.53	6.38	

M3d	Baseline	6	3.57 ± 3.46	0.06	7.19	0.25	10.16	0.463
	3d		2.34 ± 2.02	0.22	4.45	0.76	6.12	

G6h	Baseline	6	3.27 ± 0.30	1.36	7.35	3.04	10.16	0.752
	6h		4.58 ± 3.01	1.10	10.03	2.60	11.86	

G1d	Baseline	6	3.83 ± 2.16	1.56	6.10	1.53	6.56	0.753
	1d		4.10 ± 2.46	1.52	6.68	0.62	7.43	

G2d	Baseline	6	2.50 ± 0.45	1.42	4.84	1.81	6.33	0.028
	2d		5.04 ± 3.37	1.51	8.58	2.92	11.86	

G3d	Baseline	6	2.87 ± 0.75	2.08	3.65	1.81	4.05	0.225
	3d		4.58 ± 2.47	1.99	7.17	2.60	8.17	

**Table 6:** Statistics for mRNA expressions (2^−ΔΔCt^).

	*Hk2*	*P*(vs. M)	*Pfkm*	*P*(vs. M)	*Slc2a4*	*P*(vs. M)	*Prkaa1*	*P*(vs. M)	*Bdh1*	*P*(vs. M)
K	0.97 ± 0.27	—	1.43 ± 0.37	—	1.02 ± 0.27	—	1.24 ± 0.69	—	0.55 ± 0.39	—
M6h	3.02 ± 0.60	—	0.68 ± 0.42	—	0.11 ± 0.05	—	5.84 ± 0.54	—	1.32 ± 0.39	—
M1d	4.39 ± 0.84	—	1.13 ± 0.26	—	0.54 ± 0.52	—	4.79 ± 1.05	—	2.11 ± 0.30	—
M2d	2.04 ± 0.94	—	1.26 ± 0.08	—	0.76 ± 0.26	—	4.48 ± 0.73	—	0.44 ± 0.38	—
M3d	5.44 ± 1.30	—	0.60 ± 0.22	—	0.27 ± 0.15	—	4.43 ± 1.65	—	2.69 ± 0.39	—
G6h	7.14 ± 1.62	0.002	1.74 ± 0.61	0.035	0.88 ± 0.35	0.010	4.11 ± 0.43	0.009	3.22 ± 0.26	0.001
G1d	5.32 ± 0.61	0.112	0.47 ± 0.20	0.031	0.33 ± 0.26	0.518	7.35 ± 1.96	0.058	4.36 ± 0.84	0.003
G2d	4.87 ± 0.73	0.003	1.91 ± 0.59	0.098	2.05 ± 0.39	0.002	3.13 ± 0.60	0.050	3.11 ± 0.77	0.001
G3d	3.65 ± 0.48	0.036	1.27 ± 0.46	0.067	0.62 ± 0.37	0.179	3.16 ± 1.54	0.299	1.52 ± 0.39	0.011

**Table 7:** Statistics for protein expressions.

		Sample size	Mean ± SD	95% CI for mean		Minimum	Maximum	*P* (vs. K)	*P* (vs. M)
				Lower limit	Upper limit				
GLUT4	K	3	1.17 ± 10.08	0.97694	1.35640	1.100	1.250	—	—
	M6h		0.12 ± 0.00	0.11280	0.11854	0.115	0.117	<0.001	—
	G6h		1.00 ± 0.01	0.98474	1.02459	0.997	1.013	0.004	<0.001

HK2	K	3	0.13 ± 0.02	0.08252	0.17282	0.113	0.148	—	—
	M6h		0.50 ± 0.03	0.42578	0.56756	0.469	0.526	<0.001	—
	G6h		1.20 ± 0.03	1.11872	1.27528	1.165	1.228	<0.001	<0.001

PFKM	K	3	1.01 ± 0.30	0.26847	1.75887	0.675	1.246	—	—
	M6h		0.21 ± 0.03	0.12963	0.28903	0.176	0.240	0.002	—
	G6h		0.84 ± 0.11	0.57297	1.09769	0.722	0.931	0.281	0.006

PKM	K	3	0.56 ± 0.09	0.32940	0.79526	0.486	0.667	—	—
	M6h		0.86 ± 0.21	0.35194	1.37406	0.668	1.078	0.052	—
	G6h		1.14 ± 0.14	0.80526	1.47941	1.011	1.282	0.003	0.066

LDHA	K	3	0.79 ± 0.16	0.38233	1.19501	0.628	0.955	—	—
	M6h		0.66 ± 0.10	0.39943	0.91124	0.564	0.767	0.248	—
	G6h		1.25 ± 0.11	0.98714	1.52153	1.141	1.355	0.004	0.001

p-AMPK*α*	K	3	0.74 ± 0.12	0.44514	1.03886	0.622	0.861	—	—
	M6h		0.74 ± 0.02	0.70096	0.77904	0.726	0.757	0.977	—
	G6h		0.50 ± 0.08	0.31306	0.68961	0.443	0.587	0.012	0.012

p-mTOR	K	3	0.64 ± 0.10	0.38529	0.89671	0.547	0.751	—	—
	M6h		0.69 ± 0.17	0.26839	1.10827	0.517	0.855	0.702	—
	G6h		1.06 ± 0.15	0.67655	1.43678	0.905	1.211	0.012	0.021

p-4EBP1	K	3	0.93 ± 0.00	0.92154	0.92913	0.924	0.927	—	—
	M6h		0.93 ± 0.04	0.83214	1.02386	0.888	0.965	0.958	—
	G6h		0.80 ± 0.10	0.55561	1.03505	0.699	0.892	0.038	0.035

## Data Availability

All the data generated or analyzed during this study are available upon reasonable request from the corresponding author.

## Supplementary Materials

Supplementary Materials The ARRIVE checklist, the CONSORT checklist, and the raw data analyzed in this study are included in the supplemental files S1, S2, and S3.
