# Supplementary material for: Scraping Therapy Improved Muscle Regeneration through Regulating GLUT4/Glycolytic and AMPK/mTOR/4EBP1 Pathways in Rats with Lumbar Multifidus Injury
Source: Pain Res Manag. 2023 Jun 22;2023:8870256. doi: 10.1155/2023/8870256 (PMC10310458; doi:10.1155/2023/8870256)
Supplement: Supplementary Materials — The ARRIVE checklist, the CONSORT checklist, and the raw data analyzed in this study are included in the supplemental files S1, S2, and S3. [file 8870256.f1.zip › 8870256.f1/S3 raw data.docx]

Blank Group

| Rats | 1 | 2 | 3 | 4 | 5 | 6 |
| --- | --- | --- | --- | --- | --- | --- |
| Baseline temperature | 37.40 | 37.10 | 38.00 | 38.40 | 38.60 | 39.10 |
| Tactile allodynia (L/R) | 11.86 | 10.16 | 6.33 | 6.33 | 5.92 | 11.86 |
|  | 9.74 | 11.86 | 4.73 | 7.86 | 11.45 | 10.16 |
| CSA | 1932.87 | 2070.56 | 2203.68 | 1833.05 | 2252.43 | 2307.68 |

M6h Group

| Rats | 1 | 2 | 3 | 4 | 5 | 6 |
| --- | --- | --- | --- | --- | --- | --- |
| Baseline temperature | 36.70 | 37.20 | 36.10 | 36.90 | 36.60 | 36.30 |
| 6h temperature | 36.60 | 36.70 | 37.20 | 37.00 | 38.60 | 36.60 |
| Tactile allodynia (L/R) | 4.73 | 3.43 | 4.04 | 5.92 | 7.30 | 7.86 |
|  | 4.85 | 2.18 | 6.03 | 7.86 | 6.84 | 3.66 |
| 6h tactile allodynia (L/R) | 4.73 | 3.09 | 3.79 | 4.73 | 7.30 | 6.84 |
|  | 6.12 | 2.18 | 3.09 | 6.12 | 2.60 | 4.19 |
| CSA | 1331.46 | 1568.19 | 1282.48 | 1312.76 | 1220.39 | 1088.39 |

M1d Group

| Rats | 1 | 2 | 3 | 4 | 5 | 6 |
| --- | --- | --- | --- | --- | --- | --- |
| Baseline temperature | 38.40 | 39.80 | 37.30 | 37.30 | 36.40 | 38.50 |
| 1d temperature | 37.40 | 38.20 | 39.10 | 38.60 | 38.40 | 38.90 |
| Tactile allodynia (L/R) | 7.30 | 4.19 | 3.66 | 3.66 | 2.56 | 4.85 |
|  | 6.03 | 7.43 | 4.73 | 4.05 | 2.60 | 1.15 |
| 1d tactile allodynia (L/R) | 7.30 | 4.19 | 3.79 | 1.92 | 1.19 | 6.12 |
|  | 6.33 | 6.55 | 4.73 | 1.81 | 3.09 | 3.04 |
| CSA | 1476.23 | 1332.26 | 1307.14 | 1686.45 | 1946.85 | 1411.51 |

M2d Group

| Rats | 1 | 2 | 3 | 4 | 5 | 6 |
| --- | --- | --- | --- | --- | --- | --- |
| Baseline temperature | 39.30 | 39.00 | 37.00 | 37.20 | 37.50 | 36.50 |
| 2d temperature | 36.20 | 36.50 | 39.40 | 39.10 | 38.80 | 38.70 |
| Tactile allodynia (L/R) | 6.03 | 2.56 | 2.56 | 4.85 | 2.60 | 4.73 |
|  | 3.79 | 2.60 | 3.09 | 4.05 | 3.09 | 4.54 |
| 2d tactile allodynia (L/R) | 0.96 | 3.25 | 5.92 | 6.98 | 1.81 | 3.64 |
|  | 1.81 | 2.20 | 4.04 | 6.38 | 3.09 | 1.53 |
| CSA | 1227.80 | 1284.44 | 2042.97 | 1446.21 | 1381.11 | 2400.42 |

M3d Group

| Rats | 1 | 2 | 3 | 4 | 5 | 6 |
| --- | --- | --- | --- | --- | --- | --- |
| Baseline temperature | 37.30 | 41.10 | 40.60 | 37.30 | 36.90 | 37.30 |
| 3d temperature | 38.00 | 38.50 | 37.60 | 38.10 | 38.70 | 38.30 |
| Tactile allodynia (L/R) | 2.56 | 4.85 | 4.73 | 1.81 | 2.56 | 2.92 |
|  | 3.09 | 3.09 | 10.16 | .25 | 1.37 | 3.43 |
| 3d tactile allodynia (L/R) | 1.19 | 3.09 | 4.73 | 1.28 | 3.25 | 2.60 |
|  | 1.53 | 6.12 | 3.09 | 0.76 | 1.15 | 1.37 |
| CSA | 1067.95 | 1851.86 | 1335.87 | 1559.30 | 2028.83 | 1383.38 |

G6h Group

| Rats | 1 | 2 | 3 | 4 | 5 | 6 |
| --- | --- | --- | --- | --- | --- | --- |
| Baseline temperature | 38.70 | 38.50 | 37.60 | 37.40 | 37.40 | 37.60 |
| Instant temperature | 39.20 | 38.60 | 39.20 | 39.40 | 39.20 | 38.50 |
| 6h temperature | 37.90 | 40.00 | 39.10 | 39.10 | 38.80 | 38.30 |
| Tactile allodynia (L/R) | 2.56 | 11.86 | 11.86 | 2.56 | 3.25 | 2.15 |
|  | 3.09 | 10.16 | 3.09 | 3.66 | 3.09 | 3.04 |
| 6h tactile allodynia (L/R) | 3.09 | 6.12 | 6.03 | 1.15 | 0.96 | 0.96 |
|  | 2.60 | 11.86 | 10.16 | 3.09 | 2.60 | 3.09 |
| CSA | 1677.83 | 1179.66 | 2042.25 | 2020.13 | 1269.83 | 2103.44 |

G1d Group

| Rats | 1 | 2 | 3 | 4 | 5 | 6 |
| --- | --- | --- | --- | --- | --- | --- |
| Baseline temperature | 38.40 | 38.90 | 38.90 | 38.00 | 37.30 | 37.30 |
| Instant temperature | 39.80 | 38.00 | 38.70 | 40.00 | 38.60 | 39.40 |
| 1d temperature | 36.70 | 36.80 | 35.10 | 38.20 | 37.90 | 38.10 |
| Tactile allodynia (L/R) | 3.09 | 4.85 | 5.57 | 4.85 | 3.66 | 3.79 |
|  | 6.12 | 3.09 | 4.05 | 1.53 | 1.63 | 6.56 |
| 1d tactile allodynia (L/R) | 3.25 | 7.30 | 7.79 | 3.09 | 7.30 | 1.92 |
|  | 3.09 | 6.33 | 7.43 | 3.09 | 4.05 | .62 |
| CSA | 1697.55 | 1870.45 | 2283.20 | 1950.04 | 1979.17 | 2444.86 |

G2d Group

| Rats | 1 | 2 | 3 | 4 | 5 | 6 |
| --- | --- | --- | --- | --- | --- | --- |
| Baseline temperature | 38.90 | 38.90 | 38.20 | 37.60 | 37.40 | 38.50 |
| Instant temperature | 40.80 | 41.20 | 40.10 | 39.10 | 39.80 | 39.10 |
| 2d temperature | 36.00 | 35.20 | 36.30 | 39.00 | 38.80 | 38.60 |
| Tactile allodynia (L/R) | 1.15 | 2.56 | 1.38 | 2.56 | 7.43 | 4.05 |
|  | 1.81 | 3.09 | 2.56 | 6.33 | 2.80 | 2.18 |
| 2d tactile allodynia (L/R) | 4.04 | 1.92 | 3.43 | 7.86 | 2.60 | 9.74 |
|  | 2.92 | 3.66 | 4.37 | 3.66 | 3.79 | 11.86 |
| CSA | 1418.99 | 1639.49 | 1577.63 | 1674.05 | 1544.05 | 1863.33 |

G3d Group

| Rats | 1 | 2 | 3 | 4 | 5 | 6 |
| --- | --- | --- | --- | --- | --- | --- |
| Baseline temperature | 37.00 | 40.00 | 40.00 | 36.40 | 37.20 | 37.10 |
| Instant temperature | 38.80 | 38.60 | 40.20 | 38.90 | 39.70 | 38.90 |
| 3d temperature | 38.30 | 36.40 | 36.90 | 38.30 | 38.80 | 38.00 |
| Tactile allodynia (L/R) | 2.99 | 4.73 | 2.60 | 3.09 | 4.73 | 1.53 |
|  | 4.05 | 1.81 | 3.09 | 3.09 | 2.60 | 2.56 |
| 3d tactile allodynia (L/R) | 8.17 | 3.25 | 11.45 | 3.43 | 2.60 | 3.25 |
|  | 2.60 | 3.25 | 8.17 | 3.09 | 7.30 | 3.09 |
| CSA | 2098.81 | 1634.55 | 1383.12 | 2407.15 | 1362.42 | 1265.50 |

expressions of mRNA

| Rats | K | | | M6h | | | G6h | | |
| --- | --- | --- | --- | --- | --- | --- | --- | --- | --- |
|  | 1 | 2 | 3 | 1 | 2 | 3 | 1 | 2 | 3 |
| Bdh1 | 1.00 | 0.27 | 0.38 | 0.93 | 1.32 | 1.72 | 3.15 | 3.01 | 3.51 |
| Hk2 | 1.00 | 0.68 | 1.22 | 2.71 | 3.71 | 2.63 | 1.23 | 1.17 | 1.20 |
| Pfkm | 1.00 | 1.69 | 1.59 | 1.01 | 0.20 | 0.81 | 0.93 | 0.72 | 0.85 |
| Prkaa1 | 1.00 | 0.71 | 2.01 | 5.45 | 6.45 | 5.60 | 3.66 | 4.15 | 4.51 |
| Slc2a4 | 1.00 | 0.76 | 1.30 | 0.11 | 0.06 | 0.16 | 0.86 | 1.23 | 0.54 |

| Rats | K | | | M1d | | | G1d | | |
| --- | --- | --- | --- | --- | --- | --- | --- | --- | --- |
|  | 1 | 2 | 3 | 1 | 2 | 3 | 1 | 2 | 3 |
| Bdh1 | 1.00 | 0.27 | 0.38 | 2.07 | 2.42 | 1.82 | 4.88 | 3.39 | 4.80 |
| Hk2 | 1.00 | 0.68 | 1.22 | 4.72 | 5.00 | 3.43 | 6.02 | 5.03 | 4.92 |
| Pfkm | 1.00 | 1.69 | 1.59 | 0.87 | 1.11 | 1.39 | 0.33 | 0.70 | 0.38 |
| Prkaa1 | 1.00 | 0.71 | 2.01 | 5.18 | 5.58 | 3.60 | 8.57 | 5.09 | 8.40 |
| Slc2a4 | 1.00 | 0.76 | 1.30 | 0.05 | 1.08 | 0.48 | 0.47 | 0.49 | 0.02 |

| Rats | K | | | M2d | | | G2d | | |
| --- | --- | --- | --- | --- | --- | --- | --- | --- | --- |
|  | 1 | 2 | 3 | 1 | 2 | 3 | 1 | 2 | 3 |
| Bdh1 | 1.00 | 0.27 | 0.38 | 0.23 | 0.20 | 0.88 | 2.24 | 3.68 | 3.42 |
| Hk2 | 1.00 | 0.68 | 1.22 | 1.43 | 1.56 | 3.12 | 4.09 | 4.95 | 5.55 |
| Pfkm | 1.00 | 1.69 | 1.59 | 1.32 | 1.30 | 1.17 | 2.14 | 1.24 | 2.35 |
| Prkaa1 | 1.00 | 0.71 | 2.01 | 4.39 | 3.79 | 5.25 | 3.00 | 3.78 | 2.61 |
| Slc2a4 | 1.00 | 0.76 | 1.30 | 0.96 | 0.84 | 0.47 | 1.87 | 1.78 | 2.49 |

| Rats | K | | | M3d | | | G3d | | |
| --- | --- | --- | --- | --- | --- | --- | --- | --- | --- |
|  | 1 | 2 | 3 | 1 | 2 | 3 | 1 | 2 | 3 |
| Bdh1 | 1.00 | 0.27 | 0.38 | 2.40 | 3.14 | 2.53 | 1.96 | 1.21 | 1.39 |
| Hk2 | 1.00 | 0.68 | 1.22 | 4.11 | 6.71 | 5.49 | 3.18 | 3.62 | 4.14 |
| Pfkm | 1.00 | 1.69 | 1.59 | 0.84 | 0.41 | 0.55 | 1.00 | 1.10 | 1.68 |
| Prkaa1 | 1.00 | 0.71 | 2.01 | 2.53 | 5.53 | 5.23 | 4.21 | 1.40 | 3.88 |
| Slc2a4 | 1.00 | 0.76 | 1.30 | 0.37 | 0.34 | 0.10 | 0.29 | 1.02 | 0.55 |

expressions of proteins

| Rats | K | | | M6h | | | G6h | | |
| --- | --- | --- | --- | --- | --- | --- | --- | --- | --- |
|  | 1 | 2 | 3 | 1 | 2 | 3 | 1 | 2 | 3 |
| PKM | 0.67 | 0.49 | 0.53 | 1.08 | 0.67 | 0.84 | 1.28 | 1.01 | 1.13 |
| 4EBP1 | 1.00 | 0.86 | 0.94 | 1.30 | 0.87 | 1.01 | 1.09 | 1.09 | 1.08 |
| LDHA | 0.96 | 0.63 | 0.78 | 0.77 | 0.56 | 0.64 | 1.36 | 1.14 | 1.27 |
| p-mTOR | 0.55 | 0.75 | 0.63 | 0.52 | 0.86 | 0.69 | 0.91 | 1.21 | 1.05 |
| mTOR | 1.51 | 0.82 | 1.24 | 0.98 | 1.08 | 1.10 | 1.51 | 0.82 | 1.24 |
| PFK1 | 1.25 | 0.68 | 1.12 | 0.24 | 0.18 | 0.21 | 1.25 | 0.68 | 1.12 |
| HK2 | 0.15 | 0.11 | 0.12 | 0.53 | 0.47 | 0.50 | 0.15 | 0.11 | 0.12 |
| p-4EBP1 | 0.93 | 0.92 | 0.93 | 0.97 | 0.89 | 0.93 | 0.89 | 0.70 | 0.80 |
| p-AMPKα | 0.62 | 0.86 | 0.74 | 0.76 | 0.73 | 0.74 | 0.44 | 0.47 | 0.59 |
| GLUT4 | 1.25 | 1.10 | 1.15 | 0.12 | 0.12 | 0.12 | 1.01 | 1.00 | 1.00 |
| AMPKα | 0.80 | 0.66 | 0.74 | 1.59 | 0.97 | 1.24 | 0.77 | 0.61 | 0.70 |
